# Long Term Effect of Land Reclamation from Lake on Chemical Composition of Soil Organic Matter and Its Mineralization

**DOI:** 10.1371/journal.pone.0099251

**Published:** 2014-06-06

**Authors:** Dongmei He, Honghua Ruan

**Affiliations:** Faculty of Forest Resources and Environmental Science, and Key Laboratory of Forestry and Ecological Engineering of Jiangsu Province, Nanjing Forestry University, Nanjing, Jiangsu, China; Lakehead University, Canada

## Abstract

Since the late 1950s, land reclamation from lakes has been a common human disturbance to ecosystems in China. It has greatly diminished the lake area, and altered natural ecological succession. However, little is known about its impact on the carbon (C) cycle. We conducted an experiment to examine the variations of chemical properties of dissolved organic matter (DOM) and C mineralization under four land uses, i.e. coniferous forest (CF), evergreen broadleaf forest (EBF), bamboo forest (BF) and cropland (CL) in a reclaimed land area from Taihu Lake. Soils and lake sediments (LS) were incubated for 360 days in the laboratory and the CO_2_ evolution from each soil during the incubation was fit to a double exponential model. The DOM was analyzed at the beginning and end of the incubation using UV and fluorescence spectroscopy to understand the relationships between DOM chemistry and C mineralization. The C mineralization in our study was influenced by the land use with different vegetation and management. The greatest cumulative CO_2_-C emission was observed in BF soil at 0–10 cm depth. The active C pool in EBF at 10–25 cm had longer (62 days) mean residence time (MRT). LS showed the highest cumulative CO_2_-C and shortest MRT comparing with the terrestrial soils. The carbohydrates in DOM were positively correlated with CO_2_-C evolution and negatively correlated to phenols in the forest soils. Cropland was consistently an outlier in relationships between DOM chemistry and CO_2_-evolution, highlighting the unique effects that this land use on soil C cycling, which may be attributed the tillage practices. Our results suggest that C mineralization is closely related to the chemical composition of DOM and sensitive to its variation. Conversion of an aquatic ecosystem into a terrestrial ecosystem may alter the chemical structure of DOM, and then influences soil C mineralization.

## Introduction

Microbial respiration of soil organic matter (SOM) causes a large flux of CO_2_ to the atmosphere, and understanding the controls on this flux is a critical component of society's effort to cope with increasing atmospheric CO_2_ and climate change [1]–[2]. Many abiotic and biotic factors affect the global carbon (C) cycle, especially, human activities, such as the fossil fuel combustion and land use change [3]. Of these factors, land use changes, such as deforestation and afforestation, draining of wetlands, converting grassland to arable cropping and reclaiming lakes, directly affect microbial respiration [4]–[5]. Many studies on the influence of land use change have shown it results in drastic changes to soil C cycling [6]–[8]. Land use determines the vegetation type grown on the soil, and therefore the type of organic matter input to the ecosystem [5], [9]. Land use change can also increase microbial C mineralization, which may exacerbate the trend of global warming and other effects related to climate change [7].

In the late 1950s, reclaiming land from lakes became a common type of land use in China. China is a traditional agricultural country with a growing population, and it was deemed necessary to expand cultivated land to develop agriculture as well as feed an increasing population. Thus, after several decades more than 1.4×10^4^ km^2^ of land was reclaimed from lakes [3], [10]. Taihu Lake as one of the five largest freshwater lakes in China also experienced land reclamation. Taihu Lake is situated in the south of the Yangtze Delta among 30°55′–31°33′N and 119°55′–120°36′E and with a land area of 2.3×10^3^ km^2^. From the 1950s to 1980s, more than 160 lakes around the Taihu Lake basin were disappeared and the area of water surface had reduced by 13.6% [11]. In recent years, many studies have focused on the effects of reclamation on ecosystem. They observed that many negative consequences caused by reclaiming land from lakes, such as species decline for habitat loss, eliminating the natural buffers and flood control capacity, water pollution and eutrophication caused by agriculture measures and so on [12]. However, there are few studies about the effect of lake reclamation on the properties of soil organic matter in the terrestrial ecosystem and little is known about its impact on soil C cycling.

Solid soil organic matter must pass through the dissolved phase to be decomposed by microbes [13]–[14]. Although dissolved organic C (DOC) makes up only a small portion of total soil organic carbon (mostly less than 1%), it represents the active or labile C pool [15]–[17] and turn over more than 4000 times annually [18]. Thus, the DOM pools is closely correlated to the labile fraction of soil organic matter and is a sensitive indicator of its overall dynamics [16], [19]. Also, many studies observed the close relationships between biodegradability of DOC and C mineralization [14], [16], [20].

Land use is considered as the factor with the greatest influence on soil DOM because it determines the quantity and quality of organic matter input into the soil [5]. However, the mechanism of how land use influences dynamics of DOM is still not clear. Several authors have found that biodegradability of DOM largely depends on its chemical composition [21]–[23]. It is generally assumed that the easily degradable DOM consists mainly of simple carbohydrate monomers (i.e., glucose, fructose), low molecular organic acids (i.e., citric, oxalic, succinic acid), amino acids, amino sugars and low molecular weight proteins [13], [18]. The stable DOM fraction generally contains polyphenolic, aromatic structures and other complex macromolecules [24]. So, the chemical structure of DOM and the complexity of its molecules were considered to correlate with C mineralization. The chemical structure of DOM often described with UV and fluorescence spectroscopy [25]–[26]. Coupling and understanding of the chemical composition of DOM and C mineralization could be a useful tool for evaluating the influence of land use change on soil C dynamics.

In a previous study, Wang et al. [3] reported the labile SOC concentrations were different from each other under four types of land use from land reclaimed from Taihu Lake, China. Here we endeavored to link the C mineralization potential to the chemical makeup of DOM in soils of land that had been converted from lake sediments to forests and cropland. We hypothesized that this conversion of aquatic ecosystems to terrestrial ecosystems resulted in increased recalcitrance in chemical structure of DOM and ultimately affected potential soil C mineralization, because terrestrial organic matter is usually rich in aromatic and phenolic structures while DOM from aquatic environments has a lower content of aromatic C and more aliphatic C [27]–[28]. Our objectives were to (1) test for differences in C mineralization in lake sediments and terrestrial soils under different land use on reclaimed land; (2) compare the chemical composition of DOM in lake sediments and soils under different land use; (3) relate C mineralization and chemical properties of DOM in order to explore the role of DOM in C cycling in reclaimed soils.

## Materials and Methods

### Site description

**Figure 1:**
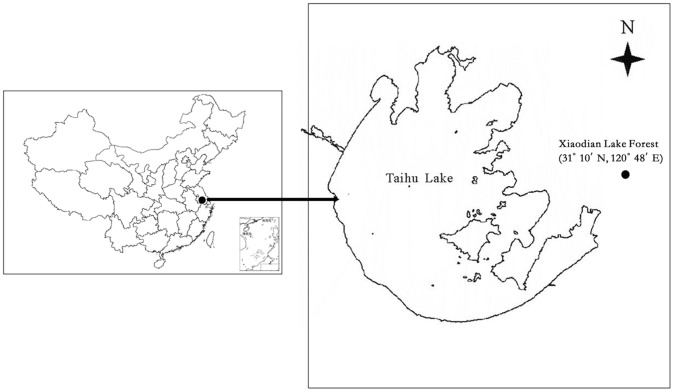
The map of study site in Xiaodian Lake area of the Taihu Lake basin China.

The study was conducted at the Xiaodian Lake Forest (31°10′N, 120°48′E), located in the northeast of Wujiang City, Jiangsu province, southeast of China (fig. 1) (The Department of Agriculture & Forestry of Jiangsu Province is the authority responsible for it. There were no specific permissions required for the study area. Our field studies did not involve endangered or protected species). The climate in this region is humid north subtropical monsoon with a mean annual temperature of 16°C, annual rainfall of approximately 1100 mm (mainly during the summer months), annual average relative humidity of 78%, and an annual non-frost period of up to 240 days [3]. This area used to be a part of the Taihu Lake, and the lake area was converted to farmland in the early 1960s. The soil properties of the reclaimed land were unsuitable for food crops and so, afforestation projects were carried out in 1969 in much of the region. After more than 40 years of forest management, it has developed into a forest park with 1.33 km^2^ forest-covered area. The dominant forest types in the park are dominated by coniferous forest (*Metasequoia glyptostroboides*), evergreen broadleaf forest (*Cinnamomum camphora*), bamboo forest (*Phyllostachys heterocycla*) and all of them are the planted tree species monoculture. Additionally, there is approximately 7.0 ha of cropland with rotations of rice and canola in parcels around the park and with the common tillage practices of ploughing, irrigation, mineral and organic fertilization. Soils in this region are all derived from lake sediment and were similar following reclamation. The general characteristics of each site were described by Wang et al. [3].

### Soil sampling

We chose four sites within coniferous forest (CF), evergreen broadleaf forest (EBF), bamboo forest (BF) and cropland (CL), respectively. Four 10 m×10 m plots were randomly established within each site. Soil samples were collected randomly using a 3 cm diameter soil sampler at 0–10 cm and 10–25 cm depths from each plot in October 2011. After removal of the litter layer, ten soil cores were taken inside each plot and pooled to form a composite soil sample. Visible roots, residues and stones were immediately removed after sampling and then field-moist soil samples were sieved through a 2 mm mesh. Soil samples were divided into two parts: one stored at 4°C for analysis of soil microbial biomass, DOM and C mineralization incubation experiments. The other part was air dried and analyzed for pH, soil total carbon (TC) and total nitrogen (TN). Lake sediments (LS) were sampled with plexiglass tube (11 cm I.D., 50 cm in length) by cylindrical sampler (Rigo Co. Φ110 mm×500 mm) at four points in the water area of the lake. Sediments in the tube were push-back and sliced at 10 cm for 0–10 cm depth and then 15 cm for 10–25 cm depth using a stainless steel spatula [29]–[30]. A total of 40 sediment samples were collected and kept in room temperature of 4°C.

### Soil physical and chemical characteristics

Soil bulk density was measured with the core method in each layer by using a 5 cm×5 cm metal cylinder [31]. Soil moisture was determined gravimetrically by oven drying at 105°C for 24 h. Soil pH was measured on air-dried soil in a 1∶2.5 (w/w) soil-water suspension using a glass electrode. Soil TC and TN were determined via dry combustion and thermal conductivity detection using an elemental analyzer (Vario EL III, Elementar, Germany). Microbial biomass carbon (MBC) was determined by chloroform fumigation as described by Vance et al. [32].

### Incubation experiment and analytical methods

#### Carbon mineralization

C mineralization was measured in each soil sample during incubation in the dark at 25°C for 360 days [33]. From each incubation sample, 100 g fresh moist soil was incubated in a 1000 ml sealed Mason jar. A 50 ml beaker containing 20 ml 1 M NaOH solution was placed in each jar to trap the evolved CO_2_. Three additional jars containing 20 ml 1 M NaOH solution but no soil were used as a control. During incubation, the evolved CO_2_ trapped in NaOH was determined by titration with 1 M HCl after precipitating the carbonate with 1 M BaCl_2_ solution. After the CO_2_ traps were taken out, the jars were left open for 2 h to maintain aerobic conditions in each jar, and resealed for further incubation. Water loss in the jars was monitored by weighing the jars and replenished by adding ultrapure water after opening [34]. The cumulative C mineralization was expressed as g CO_2_-C kg^−1^ soil.

#### DOM extraction and characterization

DOM was extracted from each soil sample before and after soil incubation. 10 mM CaCl_2_ solution was added to an aliquot of soil samples at a soil-solution ratio of 1∶2 (w/v) and shaken for 30 min in a horizontal shaker at room temperature. The suspension was spun in a centrifuge for 20 min at 4000 rpm and filtered through a 0.45 µm pore-size cellulose acetate membranes (Schleicher & Schuell, OE 67). The filtered solutions were stored frozen (−20°C) for additional analyses.

DOC concentration was determined by Shimadzu TOC-5050 total organic carbon analyzer. Hydrophilic and hydrophobic fractions in DOM were separated by Amberlite XAD-8 resin (Rohm and Haas, Philadelphia, PA) [35]. Briefly, DOM solutions were acidified to pH 2 with HCl, and then eluted through glass columns filled with Amberlite XAD-8 resin. The column effluent representing the hydrophilic fraction of DOM (Hi) was analyzed for organic C and the amount of organic C in hydrophobic fraction was calculated by the difference between organic C in DOM solution and in hydrophilic fraction. Total carbohydrates in DOM (CH) were measured by phenol-sulfuric acid method [36], using glucose as a standard. Total soluble phenolic compounds (Phe) were analyzed by Folin-Ciocalteau method [37], using tannic acid as a standard.

For spectroscopic measurements, all samples were diluted to 10 mg C L^−1^ to avoid concentration effects and were brought to a constant pH of 7.7 [21] by adding NaOH. UV absorption at 254 nm of DOM was measured using a UV/Vis spectrophotometer (Shimadzu UV-2550, Japan). The specific UV absorbance values (SUVA_254_) were determined as the ratio of the UV absorbance at 254 nm to the DOM concentration and multiplying the value of 100, i.e. (UV_254_/DOC)×100. SUVA_254_ can serve as an indicator of the aromaticity of DOM.

Fluorescence emission spectra were obtained with a Varian Cary Eclipse Fluorescence Spectrophotometer (λ_ex_ 254 nm, slit 10 nm, λ_em_ 300–480 nm, slit 10 nm, and scan speed 1200 nm min^−1^) using 1 cm cuvettes. Fluorescence efficiency index (FE) proposed by Ewald et al. [38] was considered to be proportional to the quantum efficiency and was defined as the ratio of maximum fluorescence intensity (F_max_) divided by UV absorption at the excitation wavelength of 254 nm (F_max_/Abs). The emission humification index (HIX_em_, dimensionless) was determined as the ratio between the area in the upper quarter (Σ435–480 nm) of the usable fluorescence emission spectrum and the area in the lower usable quarter (Σ300–345 nm) [39]–[41]. The HIX_em_ can show the degree of complexity and condensation of the DOM.

### Statistical analysis

The double exponential model [20], [21], [42], separating the mineralized organic C into an active C pool and slow C pool can be presented as:
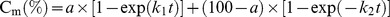
where, *t* is incubation days; C_m_ is cumulative value of mineralized C at *t* time presented as percent of initial C in the soil; *a* is the portion of organic C that is readily decomposed (in % of initial C in the soil  =  labile C); (100 − *a*) is the part of organic C that is slowly decomposed (stable C); *k_1_* and *k_2_* are mineralization rate constants for active and stable C pools (day^−1^). Mean residence time (MRT) for each pool is calculated as the reciprocal of the decomposition rate constant in the double exponential model.

This double decomposition equation for two distinct C pools with different mineralization rate constants was fitted with the nonlinear regression that was used in the Marquardt algorithm and an iterative process to find the parameter values that could minimize the residual sum of squares. The model that gave the least squared error was chosen to be the best. For a model to be chosen, all the parameters had to generate plausible results in realistic ranges. For example, the rate constants could not be negative, and the sum of active and stable C pools should not exceed initial C in the soil.

All results were reported as the mean ± SE of the four field replicates. Differences in soil properties, mineralization parameters and DOM chemical properties among land use types for each site were tested using ANOVA followed by least significant difference (LSD). T-test was used to assess the differences between soil depths. Statistical significance was determined at *p*<0.05 level. Linear regression analysis was used to determine relationships between C mineralization and chemical properties of DOM. We calculated Pearson correlation coefficients among properties of soil, C mineralization parameters and the chemical characteristics of DOM to discuss the influence of selected parameters on C mineralization. All statistical analyses were performed with SPSS 13.0.

## Results

### Soil properties

We observed variation in several soil characteristics under different land uses and soil depth (Table 1). Soil bulk density was lower at 0–10 cm soil depth than 10–25 cm depth and BF was significantly lower than all other land uses (*p*<0.05). Soil pH in all soils and sediments were ranging from 4.13 to 6.30 with the highest value in LS. TC and TN concentrations from the terrestrial soils were significantly higher at 0–10 cm soil depth (TC 14.95–35.35 g C/kg soil, TN 1.77–3.41 g N/kg soil), but both of these two factors for LS were lower at upper layer. BF soils were observed remarkably higher TC and TN than other land uses. There was significant difference in MBC and DOM between the land use types and soil depth (Table 1). MBC and DOM were significantly higher at 0–10 cm soil depth in terrestrial land uses. Under the four land uses, MBC showed highest value in CL and lowest value in CF at both soil depths, while DOM was significantly greater in CF at 0–10 cm depth and significantly lower in EBF at 10–25 cm depth. MBC and DOM in LS showed an inverse trend compared with the other land uses, which was greater at 10–25 cm depth.

**Table 1:** Soil total C concentration (TC), total N concentration (TN), dissolved organic M (DOM), microbial biomass C (MBC), pH and the bulk density of different land uses soils collected on reclaimed land from Taihu Lake, China.

Site		TC	TN	DOM	MBC	pH	Bulk density
Depth	Land use	(g C/kg Soil)	(g N/kg Soil)	(mg C/kg Soil)	(mg C/kg Soil)		(g cm^−3^)
0–10 cm	CF	19.12±1.64b*	2.24±0.15b*	126.63±2.75a*	214.50±5.77d*	4.45±0.07d*	1.22±0.03a*
	EBF	16.05±0.69bc*	1.77±0.04b*	93.50±2.56d*	370.40±9.64c*	4.78±0.08c	1.18±0.03a
	BF	35.35±1.79a*	3.41±0.22a*	110.50±2.40b*	423.01±5.13b*	4.13±0.06e*	0.89±0.06b
	CL	14.95±0.96c*	1.84±0.10b*	105.80±2.81bc*	521.98±7.72a*	5.67±0.08b*	1.16±0.03a*
	LS	16.14±1.38bc	2.26±0.23b	99.95±2.11cd*	350.03±7.64c*	6.19±0.02a	Nd
10–25 cm	CF	8.34±0.53c	1.18±0.07b	97.72±4.50b	112.77±4.43a	4.89±0.15bc	1.31±0.01a
	EBF	8.81±0.77c	1.16±0.08b	78.39±3.06c	309.94±3.90c	4.98±0.07b	1.25±0.02a
	BF	23.76±0.94a	2.59±0.25a	95.88±2.46b	232.28±4.65d	4.30±0.02c	0.99±0.06b
	CL	9.30±1.16c	1.23±0.13b	91.40±3.13b	455.99±5.20b	6.18±0.16a	1.31±0.03a
	LS	17.51±1.03b	2.34±0.13c	115.48±4.21a	558.82±10.28e	6.30±0.07a	Nd

Values are mean ± SE (n = 4). Means within a column of the corresponding depth followed by different letters are significantly different and * indicates the significant difference between the soil depth. (Significance at *p*<0.05).

CF: coniferous forest; EBF: evergreen broadleaf forest; BF: bamboo forest; CL: cropland; LS: lake sediment; Nd: Not determined.

### Chemical characteristics of DOM

The chemical composition of DOM was compared for each sample before and after incubation (Table 2). Before incubation, the fraction of CH at 0–10 cm soil depth ranged from 11.25 to 28.39% of total DOM, with the highest value in BF. The Phe at 0–10 cm soil depth ranging from 1.06 to 3.74% of total DOM was significantly larger than at 10–25 cm depth, with the highest value in CL. The Hi fraction at 0–10 cm depth was varied in the land uses with the highest in CL (61.75%) and lowest in BF (21.22%). At 10–25 cm soil depth, the fraction of CH in BF and LS was significantly larger than the other soils. The largest Phe concentration was observed in LS (3.49%). The proportion of hydrophilic C at 10–25 cm soil depth was no significant difference between each site, but it is significantly higher than at 0–10 cm depth except for CL soil.

**Table 2:** Chemical properties of soil DOM at two soil depths under different land uses reclaimed from Taihu Lake, China.

Site			SUVA_254_	FE	HIX_em_	CHPheHi		
Depth	Land use	DOM	(l mg C^−1^ m^−1^)			(% of total DOM)		
0–10 cm	CF	Initial	2.30±0.06a*	321.28±2.28b*	1.76±0.11ab	23.86±1.21b	2.50±0.04b*	32.71±1.38c*
		Δ	2.65±0.05a*	29.62±1.95c	4.17±0.11a*	32.87±2.02a*	4.68±0.22a*	12.15±2.20b
	EBF	Initial	1.00±0.09d	301.18±3.40b*	1.64±0.03b	21.83±0.49b*	2.08±0.04c	37.47±1.35b*
		Δ	2.38±0.12b*	95.03±5.61b*	4.50±0.69a*	25.40±0.51b*	2.81±0.17b*	8.72±1.91b
	BF	Initial	2.07±0.07b*	252.31±3.26d*	1.94±0.07a	28.39±1.13a	2.14±0.06c*	29.22±1.14c*
		Δ	1.05±0.06c	216.67±6.46a*	5.59±0.70a*	4.70±0.37c*	1.37±0.10c*	19.90±2.17a
	CL	Initial	1.20±0.04c*	430.40±2.29a*	1.11±0.08c*	18.98±1.02c*	3.74±0.12a*	61.75±0.70a
		Δ	0.55±0.06d*	−59.55±6.02e*	2.78±0.43b	6.09±0.37c*	−2.61±0.11e*	−3.61±1.35c*
	LS	Initial	1.15±0.06c	289.30±17.80c*	1.68±0.11b	11.25±0.72d*	1.06±0.07d*	59.58±1.19a*
		Δ	0.47±0.07d*	−4.65±1.88d	0.79±0.01c	−4.00±0.31d*	0.25±0.07d*	10.87±3.92b*
10–25 cm	CF	Initial	0.98±0.05b	253.32±1.30d	1.69±0.02a	21.37±0.46b	1.33±0.13c	47.77±1.12a
		Δ	0.60±0.07b	24.40±2.49bc	1.84±0.23c	6.74±0.17a	1.72±0.06a	7.31±1.48c
	EBF	Initial	1.20±0.06a	408.93±4.16a	1.07±0.02b	15.35±0.79c	2.18±0.09b	52.48±0.70a
		Δ	0.18±0.06c	11.25±2.46c	2.24±0.15b	2.78±0.38b	0.60±0.03a	6.98±2.83c
	BF	Initial	1.30±0.06a	170.44±2.17e	1.88±0.02a	26.14±0.81a	1.51±0.02c	46.18±0.49a
		Δ	1.10±0.04a	87.96±2.75a	2.85±0.45a	−2.14±0.85c	0.42±0.05a	17.15±0.69b
	CL	Initial	0.78±0.05c	316.91±2.46c	0.48±0.02c	11.28±0.55d	0.92±0.04d	56.06±1.65a
		Δ	0.15±0.05c	25.32±6.10b	3.52±0.16a	3.61±0.50b	−0.08±0.02c	18.33±3.58b
	LS	Initial	1.23±0.05a	350.29±11.95b	1.60±0.27a	24.81±0.37a	3.49±0.12a	40.76±1.08a
		Δ	0.20±0.04c	−48.88±11.18d	0.40±0.23d	−13.39±0.80d	0.38±0.99b	43.26±3.23a

Results shown are characteristics of initial DOM and the variation of each characteristic at the end of 360 days incubation. Values are mean ± SE (n = 4). Means within a column of the corresponding depth followed by different letters are significantly different and * indicates the significant difference between the soil depth. (Significance at *p*<0.05).

CF: coniferous forest; EBF: evergreen broadleaf forest; BF: bamboo forest; CL: cropland; LS: lake sediment; SUVA_254_: specific UV absorbance at 254 nm; FE: fluorescence efficiency (F_max_/A254); HIX_em_: humification index using emission fluorescence spectra (ratio of areas: 435–480 nm/300–345 nm); CH: carbohydrate C; Phe: phenol C; Hi: hydrophilic C; initial: properties of initial samples (soils before incubation); Δ: variation of DOM chemical properties between the initial and final value during incubation.

From the initial samples before incubation, the spectral indicators were found to be significantly different among the land uses and between soil depths (Table 2). At 0–10 cm depth, SUVA_254_ was significantly greater in CF while FE was significantly greater in CL. At 10–25 cm soil depth, SUVA_254_ and HIX_em_ were both lower in CL. Difference in FE at 10–25 cm was statistically significant between each site. There were no significant differences between soil depths in SUVA_254_ for EBF and LS, but FE for all the sites were observed significantly different between soil depths. Differences in HIX_em_ between soil depths were only found in CL.

After 360 days incubation, there were significant amounts of variation in DOM chemical properties among the land uses and lake sediments and most of the variation between soil depths was also significant (Table 2). The proportion of CH at 0–10 cm soil depth increased 17%–138% in the land uses with significantly greater value in CF. The proportion of Phe and Hi respectively increased 23%–188% and 18%–68% for CF, EBF, BF, and LS at 0–10 cm depth, but the values of these two factors for CL showed decreased trend after incubation. The increased values of Phe were significantly different from each site with the greatest variation in CF, while increased amount of Hi was lager in BF, at 0–10 cm depth. The proportion of CH fraction had the largest increase in CF, but it was observed reducing in BF and LS, at 10–25 cm soil depth. At this layer, both the proportion of Phe and Hi increased after incubation, and the largest increase was found in LS. There was no significant difference in the variation of Phe among the three forest sites. SUVA_254_ and HIX_em_ from the samples at the end of incubation were increased at both 0–10 cm and 10–25 cm soil depth (Table 2). CF had the greatest variation in SUVA_254_ and HIX_em_ at 0–10 cm depth and the variation of FE in BF was larger the other sites. There was also no significant difference in the variation of HIX_em_ among the three forest sites. The variation of FE values was significantly different between each other site and the FE values in CL and LS decreased after incubation. At 10–25 cm depth, the variation of SUVA_254_ and FE in BF was significantly larger than other sites.

### Carbon mineralization

**Figure 2:**
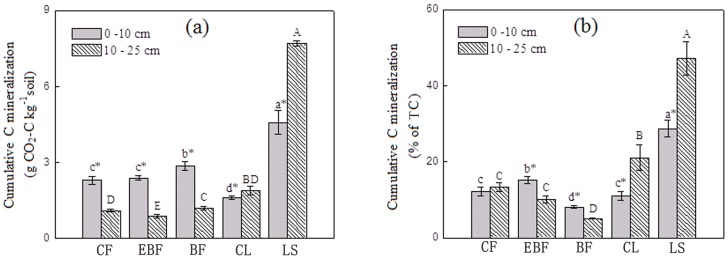
Carbon mineralized at the end of the 360 days incubation period for different land uses and lake sediments. Bars represent standard errors (n = 4). Different letters above bars indicate significant differences (*p*<0.05) of mean values of different sites. * indicates the significant difference (*p*<0.05) between the soil depth.

The cumulative CO_2_ production under different land uses and sediments ranged from 0.88 to 7.72 g CO_2_-C kg^−1^ soil and there were significant difference between soil depths (Fig. 2a). At 0–10 cm soil depth, cumulative C mineralization in BF soils (2.87 g CO_2_-C kg^−1^ soil) was significantly greater than other land uses and CL had the lowest amounts of cumulative CO_2_-C (1.62 g CO_2_-C kg^−1^ soil). The cumulative CO_2_-C for the three forest sites decreased with the increasing soil depth. At 10–25 cm soil depth, C mineralization was found to be significantly different in the following order of CL>BF>CF>EBF (Table 3). Representing the precursor of the four terrestrial soils, LS had different C mineralization patterns than the four terrestrial soils, and its evolution of cumulative CO_2_-C (4.59 and 7.72 g CO_2_-C kg^−1^ soil, respectively) was significantly larger than other soils, at 0–10 cm and 10–25 cm soil depth. After 360 days of incubation, the percentage of cumulative mineralized C (as % of TC) ranged from 5.11% to 47.19% with the highest C mineralization in LS at 10–25 cm depth (Fig. 2b). At 0–10 cm soil depth, the percentage of mineralized C was significantly different between each other site with largest value in EBF (15.23%) and lowest value in BF (8.11%). At 10–25 cm soil depth, CL had the highest percentage of cumulative mineralized CO_2_-C (21.07%) among the four land uses, approximately four times larger than BF, which had the lowest percentage of mineralized C (5.11%).

**Table 3:** C mineralization kinetics of soil after 360 days incubation at 25°C: cumulative CO_2_-C, sizes of the labile and stable C pools, mineralization rate constants and mean residence times of the labile and the stable C pools.

Site		C_m_	Labile C	Stable C	*k_1_*	*k_2_*	MRT_1_	MRT_2_	*r^2^*
Depth	Land use	(%)	(%)	(%)	(day^−1^)	(day^−1^)	(days)	(years)	
0–10 cm	CF	12.24	0.75	99.25	0.03	0.00036	30.48	7.68	0.993
	EBF	15.23	0.72	99.28	0.13	0.00048	7.82	5.80	0.979
	BF	8.11	0.20	99.80	0.10	0.00023	10.17	12.13	0.998
	CL	10.95	2.62	97.38	0.02	0.00022	53.13	12.60	0.999
	LS	28.71	1.13	98.87	0.19	0.00098	5.15	2.85	0.983
10–25 cm	CF	13.45	3.07	96.93	0.03	0.00032	33.27	8.67	0.995
	EBF	10.11	4.81	95.19	0.02	0.00016	61.71	17.80	0.999
	BF	5.11	1.42	98.58	0.02	0.00011	48.62	26.11	0.999
	CL	21.07	1.70	98.30	0.07	0.00065	13.53	4.26	0.993
	LS	47.19	7.09	92.91	0.12	0.00128	8.11	2.16	0.957

CF: coniferous forest; EBF: evergreen broadleaf forest; BF: bamboo forest; CL: cropland; LS: lake sediment; C_m_: cumulative mineralized C as percentage of initial C in the soil. Labile C: rapidly mineralizable C (calculated using a double exponential model); Stable C: slowly mineralizable C (calculated using a double exponential model); *k_1_*: mineralization rate constant of the labile C pool (double exponential model); *k_2_*: Mineralization rate constant of the stable C pool (double exponential model); MRT_1_: Mean residence times of the labile C pool (MRT_1_ = 1/*k_1_*); MRT_2_: Mean residence times of the stable C pool (MRT_1_ = 1/*k_2_*); *r^2^*: coefficient of determination of the double exponential model.

Values are mean (n = 4).

**Figure 3:**
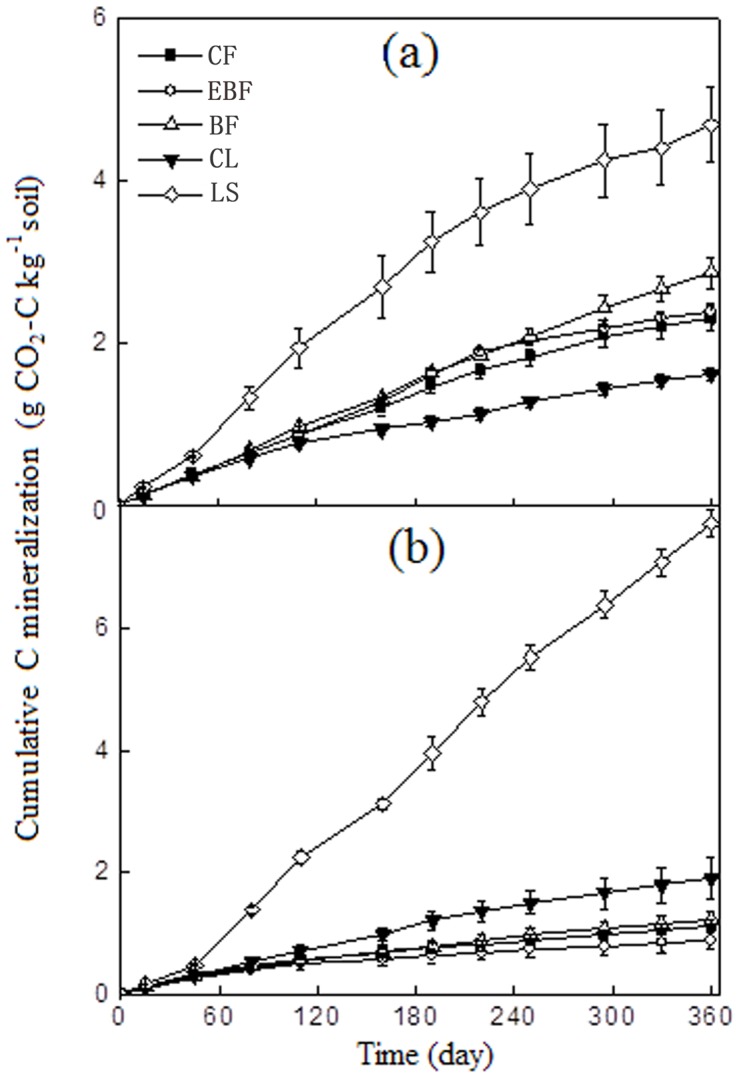
Dynamics of C mineralization of soils under different land use types reclaimed from Taihu Lake, China. (a): C mineralization at 0–10 cm depth; (b): C mineralization at 10–25 cm depth. Bars indicate the standard errors (n = 4).

The rate of CO_2_-C evolution from all land use types were highest at the beginning and then decreased progressively with the advancement of time (Fig. 3). The CO_2_-C evolution patterns in all land uses and lake sediments were best described by the double exponential model, with the model fits resulting in *r* ^2^ values between 0.96 and 0.99 (Table 3). The size of the labile C pool in different soils was comprised of 0.2%–7.09% with the highest value in LS at 10–25 cm depth. The *k_1_* values (mineralization rate constant) of labile C at 0–10 cm depth ranged from 0.02 to 0.19 with the mean residence time (MRT_1_) of 21 days (average of all soils), varying from 0.02 to 0.12 at 10–25 cm depth with an average MRT_1_ of 33 days. Determination of the size and turnover of the stable C pool showed values at 0–10 cm soil depth were larger than those at 10–25 cm soil depth (Table 3). The mineralization rate constants of the stable C pool (*k_2_*) were 2–3 orders of magnitude lower than *k_1_*, ranging from 0.00022 to 0.00098 in upper soil layer with an average of MRT_2_ of 9 years and ranging from 0.00011 to 0.00129 in lower soil layer with an average of MRT_2_ of 12 years. At 0–10 cm soil depth, the mean residence time of active and stable C pools in CL sites was larger than the others, whereas at 10–25 cm soil depth, the largest mean residence time of stable C pools was found in BF. The mean residence time of active and stable C pools in LS was lower than all other land use types.

### C mineralization in relation to DOM characteristics

**Figure 4:**
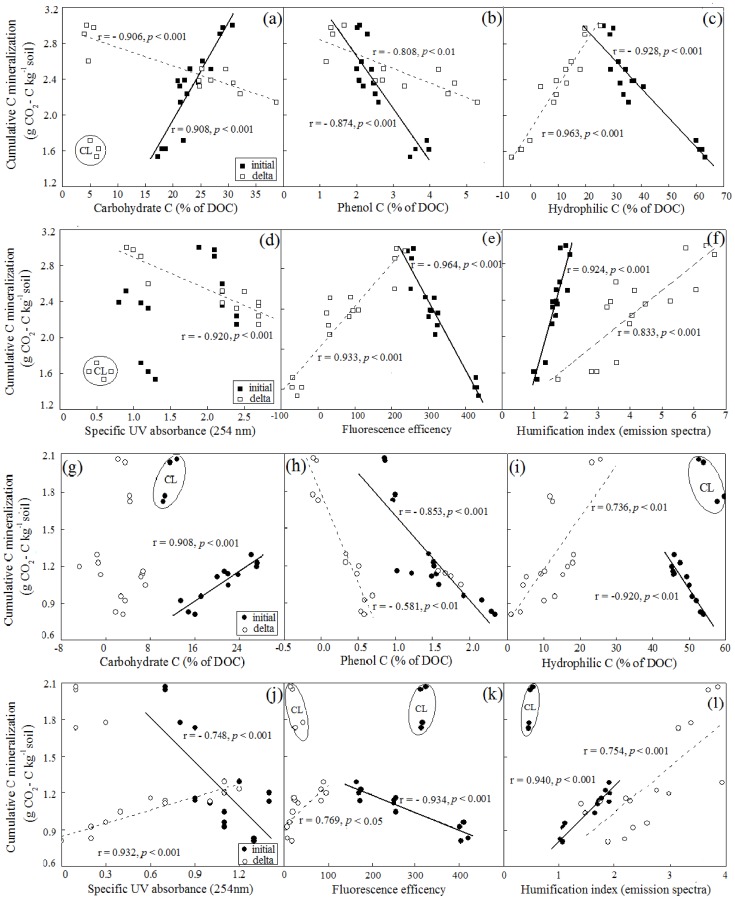
Relationships between cumulative C mineralization and DOM chemical properties of the four land use types. 0–10 cm soil depth: a–f; 10–25 cm soil depth: g–l. (a and g) between carbohydrate carbon percentages and cumulative CO_2_-C, (b and h) between phenol carbon percentages and cumulative CO_2_-C, (c and i) between hydrophilic carbon and cumulative CO_2_-C, (d and j) between SUVA_254_ values and cumulative CO_2_-C, (e and k) between the fluorescence efficiency and cumulative CO_2_-C, (f and l) between the humification index (emission fluorescence spectra) and cumulative CO_2_-C. The curves for the figures are generated with the data of the CL samples excluded.

C mineralization was significantly correlated with DOM characteristics and its change (the variation of DOM properties between beginning and ending of incubation) under the four land uses (Table 4, Fig. 4). The CO_2_-C evolution at the 0–10 cm soil depth had a positive correlation with the proportion of CH, variation of the Hi fraction (ΔHi), fluorescence efficiency variation (ΔFE) and HIX_em_ variation (ΔHIX_em_), while had a negative correlation with initial Phe, Hi, FE and variation of CH (ΔCH), Phe (ΔPhe) and SUVA_254_ (ΔSUVA_254_) (Fig. 4). The high proportion of labile C pool (*a* value of the double exponential model) from upper soil was related to the high proportion of Phe, Hi fraction and high values of FE. In contrast, it was negatively related to most of the parameters including CH fraction, ΔPhe, ΔHi, HIX_em_, SUVA_254_, ΔFE, ΔHIX_em_ and ΔSUVA_254_ (Table 4). The *k_1_* values of upper soils were correlated very well with proportion of Phe and Hi, the values of FE, HIX_em_, ΔFE and ΔHIX_em_, whereas the *k_2_* values were only correlated with Phe, ΔCH, ΔPhe and ΔSUVA_254_ (Table 4).

**Table 4:** Pearson correlation coefficients (r-value) between C mineralization and soil properties and chemical characteristics of DOM under four terrestrial land use types reclaimed from Taihu Lake, China.

		DOC	CH	ΔCH	Hi	ΔHi	Phe	ΔPhe	SUVA_254_	ΔSUVA_254_	FE	ΔFE	HIX_em_	ΔHIX_em_
0–10 cm	*a*	−0.140	−0.762**	−0.315	0.976**	−0.893**	0.951**	−0.782**	−0.504*	−0.538*	0.982**	−0.871**	−0.902**	−0.702**
	*k_1_*	−0.586*	0.373	0.048	−0.555*	0.499*	−0.792**	0.339	−0.263	0.275	−0.737**	0.752**	0.510*	0.532*
	*k_2_*	−0.285	−0.148	0.803**	−0.346	0.090	−0.548*	0.666**	−0.289	0.849**	−0.283	0.043	0.210	0.106
	MRT_1_	0.270	−0.600*	−0.203	0.822**	−0.742**	0.948**	−0.604*	−0.101	−0.447	0.928**	−0.876**	−0.755**	−0.665**
	MRT_2_	0.130	0.099	−0.886**	0.414	−0.145	0.562*	−0.762**	0.142	−0.921**	0.305	−0.031	−0.268	−0.117
	CO_2_-C	0.085	0.908**	0.044	−0.928**	0.963**	−0.873**	0.573*	0.432	0.279	−0.964**	0.934**	0.925**	0.833**
10–25 cm	*a*	−0.600*	−0.263	0.390	0.135	−0.696**	0.755**	0.368	0.200	−0.499	0.776	−0.688**	−0.954**	−0.534*
	*k_1_*	0.170	−0.653**	0.276	0.642**	0.466	−0.796**	−0.483	−0.819**	−0.456	0.090	−0.230	0.575	0.586*
	*k_2_*	−0.420	0.408	−0.383	−0.394	−0.410	0.916**	0.189	0.825**	0.235	0.172	0.125	0.079	0.459
	MRT_1_	0.142	−0.710**	0.469	0.662**	0.318	−0.758**	−0.322	−0.886**	−0.560*	0.199	−0.404	−0.700*	−0.436
	MRT_2_	−0.088	0.694**	−0.794**	−0.578*	0.028	0.581*	−0.074	0.885**	0.661**	−0.360	0.680**	0.191	−0.122
	CO_2_-C	0.310	−0.446	0.014	0.423	0.736**	−0.853**	−0.581*	−0.748**	−0.223	−0.122	0.012	0.940*	0.754**

n = 16; abbreviations see Table 2 and Table 3;

*Significance at *p*<0.05;

**Significance at *p*<0.01.

In 10–25 cm soil layer, cumulative CO_2_-C positively correlated with proportion of CH and the values of HIX_em_, ΔHi, ΔFE, ΔHIX_em_, ΔSUVA_254_, while negatively correlated with proportion of Phe and Hi, as well as the values of FE and SUVA_254_ (Table 4). The proportion of labile C pool showed positive correlations with Phe and FE, and showed the negative relationships with ΔHi, HIX_em_ and ΔFE. The *k_1_* values highly related to proportion of CH, Phe and Hi, as well as values of SUVA_254_ and ΔHIX_em_. The relationships between *k_2_* values and the properties of DOM were similar with the *k_1_* values (Table 4).

## Discussion

### Chemical characteristics of DOM

The decomposition rate of organic matter is in part controlled by its chemical fractions and structures [13], [21]. The highest portion of CH fraction in BF was due to a lot of litter and root exudate input. For the four land uses, the percentage of CH was observed lower in CL, which may be explained by two reasons: (i) soil microbial community may deplete CH fraction to a greater degree because fertilization can increase the microorganism activity [9] and (ii) the type of crop residue input may influence the chemical composition of DOM [9]. Different from the land uses, the CH for LS was greater at 10–25 cm, probably due to the deeper sediments in anoxic condition would limit the microbial activity [43]. The lowest proportion of phenol C of the DOM from the un-incubated soils was found in LS at 0–10 cm soil depth, possibly because most of the DOM is derived from algae, bacteria and macrophytes, which contain lower phenolic groups [44]. The highest percentage of Hi fraction in CL agreed with Chantigny [5] who reported that DOM from crop residue contained more hydrophilic fractions. During the incubation period, the quality and quantity of organic matter in soils changed, which should lead to variation in the chemical structure of DOM. The proportion of Hi fraction, CH and Phe in DOM under most of the sites were observed to increase at the end of incubation, indicating that some insoluble components were converted to soluble fractions, possibly due to desorption of adsorbed organic matter or other biotic regulatory mechanisms [13], [45]. Kalbitz et al. [24] reported that relative increase in polysaccharides after incubation is likely caused by microbial formation and that many bacteria and fungi can release diverse polysaccharides. The relative enrichment in phenols was possibly due to oxidation and fragmentation of lignin in soils [46]. Although both of the percentage of labile and stable fractions in DOM increased at the end of the incubation due to complement of soil organic matter [20], there was a greater increase in the proportion of Phe than CH and Hi fractions. The larger increase in the proportion of Phe at the end of incubation suggested that phenols were the most stable fractions, even though these compounds can experience partial degradation [47]. Our results suggested that CH and Hi fractions were preferentially utilized by microorganisms while phenols were resistant to degradation during incubation. Unlike the other sites, both the proportion of Phe and Hi fractions in CL DOM decreased slightly, probably due to the influence of management practices such as fertilization which changed the soil environment and accelerated both of them degradation [9], [48].

The specific UV absorbance at 254 nm (SUVA_254_), humification indices (HIX_em_) and fluorescence efficiency indices (FE) have been used to characterize the content of aromatic structures and complexity degree in DOM [49]–[51]. In our study, SUVA_254_ and HIX_em_ decreased with soil depth, consistent with Corvasce et al. [52] and Bu et al. [53], suggesting that the aromatic fraction of partially degraded lignin-derived compounds might be gradually adsorbed by upper mineral soil and protected from microbial degradation [54]. CF and BF soils had the highest SUVA_254_ and HIX_em_ values, indicating that there were more complex and condensed polyaromatic structures in the DOM due to the presence of ligninolitic compounds [55]. Our results agree with Khomutova et al. [56] who also found values of specific UV absorbance at 260 nm were larger in coniferous forest soils compared to deciduous forest and pasture soils, suggesting that DOM under conifer soils is enriched in hydrophobic aromatic compounds [57]. Just like SUVA_254_ and HIX_em_, fluorescence efficiency (FE) was also used to express the degree of condensation of DOM. The low FE in BF soils may be partially attributed to its highly substituted aromatic structural features and its inter- or intra-molecular bonding which could result in self-quenching within macromolecules [26]. Ewald et al. [38] drew a similar conclusion that fluorescence efficiency had an inverse linear correlation with molecular weight of the fulvic acid fractions, which was due to internal quenching in macromolecules caused by energy transfers. The smaller values of SUVA_254_ and HIX_em_ as well as larger value of FE were in CL consistent with Chantigny [5] who reported there were more lignin and other recalcitrant compounds in forest litter compared to crop residues. The increase of SUVA_254_ and HIX_em_ under different sites suggested aromatic structures were accumulating with the decomposition of the labile components of DOM during incubation [21], [50]. Hur et al. [27], [44] also found the increase of SUVA values for all the DOM samples after microbial incubation and attributed this result to preferential microbial utilization of non-aromatic fractions and/or to microbial transformation of labile compounds into aromatic C structures. At 0–10 cm soil depth, the increase of SUVA_254_ and HIX_em_ in CL were significantly smaller than the forest soils, indicating the same conclusion that DOM in CL contains smaller complex compounds. Although the changes in spectra parameters during incubation exhibited increasing trends under most land use types, the increased DOM for LS was smaller than other land uses. This result suggests that the accumulation of condensed aromatic structures was relatively small during incubation, supporting the previous statement that accumulation of phenols was small compared to hydrophilic fractions for LS DOM. For the soil DOM samples, a pronounced increase in FE value was inconsistent with the changes in HIX_em_ or SUVA_254_, which couldn't be explained by fluorescent quenching. This inconsistency may be attributed to the characterization method of FE influenced by the changes in DOM properties.

### Soil carbon mineralization in relation to DOM characteristics

The C mineralization was significantly higher at 0–10 cm soil depth regardless of vegetation types for all land uses except CL. The higher C mineralization observed at 10–25 cm soil depth in CL was likely due to the tillage practice of ploughing which stimulated the C mineralization in deeper layers [58]. At 0–10 cm soil depth, cumulative C mineralization in BF soils was greater than other land uses, also possibly due to plenty of SOC deriving from degradation of bamboo litter and rhizomatous. While cumulative C emission for CL was significantly lower than the forest soils at 0–10 cm and Zhao et al. [20] observed the similar result that the average cumulative mineralized CO_2_-C in forest sites and arable sites was 382 and 279 mg C/kg soil, respectively. The amount of cumulative mineralized CO_2_-C during 360 days incubation was significantly higher in lake sediments compared to the terrestrial soils. This result was consistent with our primary hypothesis. Mora et al. [33] observed that during 10 days incubation 0.5% of the sediment C, as well as 0.25% of soil C was mineralized.

The analysis of double exponential model showed that emission of CO_2_-C increased as the labile C pool (*a*) decreased, which was inconsistent with the results of Kalbitz et al. [21] and Bu et al. [59]. During the incubation period, lake sediments had a higher percentage of C mineralized (as % of TC), a larger pool of labile C, and shorter residence times for labile and stable C. This suggested that lake sediments were enriched in less complex, more easily mineralized forms of C [60]–[62]. The smaller fraction of mineralized C and lower mineralization rate constants for the stable C pool found in BF soils suggested that organic C in BF soils was composed mainly of polyphenols and aromatic structures deriving from the decomposition of bamboo rhizomes and recalcitrant to biodegradation [63]–[65]. Compared to forest soils, both labile and stable C pools in CL soils had lower mineralization rate constants, which was different from the result of Kalbitz et al. [21]. This discrepancy was likely explained by DOM representing a small fraction of labile C in CL soils, and mineralization of soil C can't be explained only by consumption of DOM. Zhao et al. [20] also found no correlations between the decrease in quantity of DOM and soil C mineralization. At 0–10 cm depth, mineralization rate constants for both the labile and stable C pool of CF soils were significantly smaller than EBF soils, consistent with previous studies which found C mineralization rate in a conifer forest to be lower than in an evergreen broadleaf forest [66].

In our study, we found the chemical compositions and spectra characteristics of DOM were correlated with C mineralization, but the relationship varied across land uses. The cumulative CO_2_-C emission showed a significant positive correlation with proportion of CH at the beginning of the incubation and a significant negative relationship with the proportion of initial Phe. The results suggest that CH and Phe respectively represent the labile and refractory fractions in DOM, and they may regulate the mineralization potential of SOM. Soil carbohydrates are readily available for biodegradation and often have short half-lives due to rapid uptake and assimilation by soil microbial communities [22]. At the end of incubation, the increase in proportion of CH at 0–10 cm soil depth had a negative relationship with cumulative CO_2_-C emission, similar to Tian et al. [34], and emphasizing that carbohydrates are utilized preferentially by microorganisms [13], [21]. With the consumption of labile fractions, the increasing proportion of Phe was closely related to the less CO_2_-C emission, explained by inhibiting the activity of various enzymes [34], [46]. The correlation between ΔCH (0–10 cm), ΔPhe (0–10 cm), CH (10–25 cm) and cumulative CO_2_-C emission in CL statistically deviated from the regression line (fig. 4a, 4b and 4g), suggesting that the contribution rate to C mineralization made by Phe or CH in CL was different from other land uses. Hi fractions of DOM separated by XAD-8 resin can be characterized as labile soluble organic moieties, particularly carbohydrates, amino sugars and low-molecular-weight organic acids and show a higher degree of biodegradation [13], [23], [35], [57]. In our study, the increase of Hi fraction was strongly tied to increasing C mineralization, which was inconsistent with the general results of previous studies [21], [44], [67]. This result attributed to the fact that part of the hydrophilic fractions such as hydrophilic neutral (HiN) is composed mainly of carbohydrates that are bound to aromatic compounds and are converted into typical hydrophobic fractions during long term incubation [21]. However, the correlation of Hi and C mineralization at 10–25 cm depth in CL also deviated from the regression line.

Recently, fluorescence and UV spectroscopy has been used successfully to describe the chemical properties of DOM [21], [53], [68]–[69]. In many studies, the extent of C mineralization was inversely related to the specific UV absorbance of DOM [13], [53], [70]–[71]. This was confirmed by our results observed in 10–25 cm soil depth, indicating that UV-inactive substances were degraded preferentially. The relative increase in SUVA_254_ at the end of incubation was positively related to cumulative CO_2_-C emission, which suggests that more aromatic structures were cumulated during the C mineralization. Fluorescence spectroscopy can provide additional information relating to structure, functional groups, conformation, and heterogeneity, as well as dynamic properties of DOM [25]–[26], [49]. In our study, the FE and HIX_em_ deduced from fluorescence emission spectra exhibited strong correlation with CO_2_ emission. The FE and HIX_em_ for initial DOM respectively showed negative and positive correlation with C mineralization, suggesting that in the long-term incubation not only labile C but also stable C contributed to the C mineralization. The ΔFE and ΔHIX_em_ during incubation were positively correlated with C mineralization, supporting expectation that with the oxidation or degradation of labile fractions, more aromatic compounds and other complex stable molecules are accumulated [68]. The relationship between part of spectroscopy parameters and cumulative CO_2_ emission in CL also deviated from the regression line (fig. 4d, 4k and 4l) possibly due to the effluence of land use and management.

## Conclusions

Soils from varied land uses under reclaimed area from Taihu Lake had significant differences in soil DOM chemical properties and C dynamics. The C mineralization of lake sediments was much larger than that in terrestrial ecosystems, confirming our hypothesis. For the terrestrial soils, C mineralization in the upper soil layer was higher in BF and lower in CL. We have found that C mineralization in our study was fitting the double exponential model and the kinetics parameters were closely related to the chemical properties of DOM and sensitive to its variation. Cumulative CO_2_-C was positively correlated with the carbohydrates while negatively correlated with phenols and the aromaticity of DOM, indicating labile compounds are preferentially utilized by microbes. Moreover, the variation of Phe (ΔPhe) and humificaton (ΔHIX_em_) at the end of incubation showed reverse correlation with cumulative CO_2_, suggesting in the long-term incubation stable compounds also make contribution to C mineralization.

Overall, our study suggests that in conversion from aquatic ecosystem to terrestrial ecosystem, different land use types with different vegetation cover and varied management practices lead to significant changes in the chemical structure of DOM, which can influence the C dynamics. Inconsistent trend observed in CL soils may be attributed to the tillage practices (e.g. fertilization, crop rotation).
